# RANKL Expression in Periodontal Disease: Where Does RANKL Come from?

**DOI:** 10.1155/2014/731039

**Published:** 2014-02-27

**Authors:** Bin Chen, Wenlei Wu, Weibin Sun, Qian Zhang, Fuhua Yan, Yin Xiao

**Affiliations:** ^1^Institute and Hospital of Stomatology, Nanjing University Medical School, 30 Zhongyang Road, Nanjing, Jiangsu 210008, China; ^2^Bone Research Laboratory, Institute of Health and Biomedical Innovation, Queensland University of Technology, Kelvin Grove Campus, Brisbane, Qld 4059, Australia

## Abstract

Periodontitis is an inflammatory disease characterized by periodontal pocket formation and alveolar bone resorption. Periodontal bone resorption is induced by osteoclasts and receptor activator of nuclear factor-**κ**B ligand (RANKL) which is an essential and central regulator of osteoclast development and osteoclast function. Therefore, RANKL plays a critical role in periodontal bone resorption. In this review, we have summarized the sources of RANKL in periodontal disease and explored which factors may regulate RANKL expression in this disease.

## 1. Introduction

Periodontitis is an inflammatory disease characterized by periodontal pocket formation and alveolar bone resorption, and it is one of the most common chronic inflammatory diseases in aged populations. *Porphyromonas gingivalis* (*P. gingivalis*), *Actinobacillus actinomycetemcomitans* (*A. actinomycetemcomitans*), and *Treponema denticola* (*T. denticola*) are major periodontal pathogens involved in various forms of periodontitis; however, simply the variety and count cannot determine the type or severity of periodontitis, which indicated that immune responses against periodontal pathogens may greatly affect the course of periodontal diseases, but the mechanisms of periodontal bone resorption remain to be established [1].

Periodontal bone resorption is induced by osteoclasts. A balance between bone resorption by osteoclasts and bone formation by osteoblasts determines the level of bone mass. Receptor activator of nuclear factor-*κ*B ligand (RANKL), its receptor RANK, and a decoy receptor osteoprotegerin (OPG) are key molecules in regulating osteoclast differentiation, recruitment, and function [2, 3]. RANKL is essential for the complete differentiation of osteoclast precursor cells [4] and plays a critical role in periodontal bone resorption [1]. In this review, we have summarized the sources of RANKL in periodontal disease and explored which factor may regulate RANKL expression in this disease.

## 2. RANKL Expression in Periodontal Disease

RANKL, a polypeptide of 314 amino acids, encoded by the gene TNFSF11 and expressed in a membrane-bound protein or in secreted forms [5], is a member of the TNF cytokine family and plays a very important role in periodontal bone resorption. The level of RANKL mRNA has been reported to be highest in advanced periodontitis compared with moderate periodontitis or healthy groups. In addition, upregulated RANKL levels are related to the number of *P. gingavalis*, a major periodontal bacterium, in clinically obtained periodontal tissue [6]. Later studies have demonstrated that bone resorption can be decreased by inhibiting the RANK/RANKL signal way during experimental periodontitis in rats [7, 8]. These results suggest that RANKL plays an important role in periodontal resorption and RANKL inhibition can inhibit periodontal bone resorption.

RANKL is identified in lymphocytes, stromal cells, and other types of cells [4, 9–11]. Liu et al. examined RANKL mRNA expression at the cellular level using in situ hybridization and found that RANKL mRNA was expressed in inflammatory cells, mainly lymphocytes and macrophages [11]. In addition, proliferating epithelium at the vicinity of inflammatory cells expressed high levels of RANKL mRNA [11]. Confocal microscopic analyses showed that both B cells and T cells, but not monocytes or fibroblasts, are the major cellular sources of RANKL in the bone resorptive lesion of periodontal disease [10]. However, other cells may also be an important source in this process because they can regulate RANKL expression indirectly by excreting proinflammatory cytokines, which can subsequently regulate the function of lymphocytes.

### 2.1. B and T Lymphocytes Are the Primary Sources of RANKL in the Bone Resorptive Lesion of Periodontal Disease

To determine the cellular source of RANKL in bone resorptive periodontitis, enzyme-linked immunosorbent assay (ELISA) and double-color confocal microscopic analyses have been used. Results of ELISA demonstrated that soluble RANKL (sRANKL) production was significantly elevated in gingival tissues with periodontal disease compared to healthy gingival tissues. Confocal microscopic analyses showed that both B and T cells, but not monocytes or fibroblasts, were the cellular source of RANKL in the bone resorptive lesions of periodontal disease. Despite the potential involvement of other factors in the bone destruction process, prominent expression of RANKL by B and T cells in the periodontal disease lesions seems to play a primary role in the augmentation of bone resorption processes in this disease [10]. Further study indicated that it is the activated but not naïve B and T lymphocytes that are the major sources of RANKL [10]. We will introduce the RANKL expression of lymphocytes in the following paragraph.

### 2.2. T Cells Stimulated by Periodontopathic Microorganisms Can Modulate Periodontal Bone Resorption through Upregulation of RANKL Production by an Adaptive Immune Response

Kawai et al. reported that regulation of T-lymphocyte function can affect periodontal bone resorption in periodontal disease [12–14]. Further studies indicate that T lymphocytes specific to *A. actinomycetemcomitans* are associated with periodontal disease and periodontal bone resorption in *A. actinomycetemcomitans*-infected rats occurred because RANKL production was upregulated [14–16].

T cells isolated from the gingival tissue of *A. actinomycetemcomitans*-immunized *P. pneumotropica* ^+^ mice proliferated *in vitro* and produced sRANKL in response to both *antigens* presentation. However, gingival T cells isolated from nonimmunized *P. pneumotropica* ^+^ mice did not show such a proliferative response to either *A. actinomycetemcomitans*-antigen or *P. pneumotropica*-antigen presentation by antigen-presenting cells, nor did they produce sRANKL [17]. These results may also indicate that periodontopathic microorganisms, such as *A. actinomycetemcomitans*, may induce bone resorption by stimulating T cells and then upregulating RANKL expression.

### 2.3. Different Types of CD4^+^ T Cells and RANKL Expressions in Periodontal Tissues

Since the bacteria involved in periodontal disease are extracellular pathogens, CD4^+^ T cells appear to play a major role in the antigen recognition of these bacteria components. Many studies indicated this opinion: Baker et al. have reported that bone loss decreased in CD4^+^ T cells deficient mice after oral infection by *P. gingivalis*, but no change in mice deficient in CD8^+^ T cells or NK1^+^ T cells [18]. Baker et al. demonstrated the importance of the adaptive immune response, especially CD4^+^ T cells, in the bone loss consequent to oral infection [18]. Another research also indicated that CD4^+^ T cells were the predominant cell type present in periodontitis gingival tissues, and they expressed RANKL more than dendritic cells or monocytes [19]. Uncommitted (naive) murine CD4^+^ T helper cells can be induced to differentiate into T helper 1 (Th1), Th2, and Th17 and regulatory T (Treg) phenotypes depending on the local cytokine milieu. IL-27, a member of the IL-6/IL-12 family cytokines, was found to greatly inhibit both mRANKL expression and sRANKL secretion in CD4^+^ T cells activated by T cell receptor ligation [20]. In contrast, in differentiated Th17 cells, IL-27 much less efficiently inhibited RANKL expression after restimulation [20]. Therefore, different types of T cells may play different roles in RANKL expression regulation.

#### 2.3.1. Th1, Th2 Cells, and RANKL Expression in Periodontal Tissues

In general, Th1-type cloned T-cells produce consistently higher levels of RANKL than Th2-type T-cells, and RANKL expression induced by TCR/CD28 costimulation is suppressed in the presence of IL-4, suggesting that RANKL is predominantly expressed on Th1-type T-cells [14]. In experiments carried out with rat, T-cells, CD28, and TCR stimulation could upregulate more RANKL expression than TCR or CD28 stimulation alone, indicating that costimulatory signals are necessary to maximize RANKL expression. Furthermore, Th1 polarization by IL-12, in addition to TCR/CD28 stimulation, enhanced the expression of RANKL on the T cells, whereas Th2 polarization by IL-4 reduced the RANKL expression on the same T-cells activated with TCR/CD28 stimulation. Thus, Th1-type T-cells seem to be potentiated to express RANKL as compared to Th2-type T-cells. Costimulatory double signals from TCR and CD28 are also required for the optimal expression of RANKL [14].

There is supportive, though not conclusive, evidence that Th1 cells and their cytokines characterize early/stable periodontal lesions [21]. Th1 cell cytokines, IFN-*γ* and TNF-*α*, are related to RANKL expression. IFN-*γ* is a major product of activated T helper cells that can function as a pro- or antiresorptive cytokine. IFN-*γ* blunts osteoclast formation through direct targeting of osteoclast precursors but indirectly stimulates osteoclast formation and promotes bone resorption by stimulating antigen-dependent T cell activation and T cell secretion of the osteoclastogenic factors RANKL and TNF-*α*. Analysis of the *in vivo* effects of IFN-*γ* has been tested in 3 mouse models of bone loss including ovariectomy, LPS injection, and inflammation via silencing of TGF-*β* signaling in T cells models. The results revealed that IFN-*γ* has both direct antiosteoclastogenic and indirect proosteoclastogenic properties *in vivo*. Under the conditions of estrogen deficiency, infection, and inflammation, the net balance of these two opposing forces is biased toward bone resorption. Inhibition of IFN-*γ* signaling may thus represent a novel strategy to simultaneously reduce inflammation and bone loss in common forms of osteoporosis [22].

#### 2.3.2. Th17 Cells and RANKL Expression in Periodontal Tissues

In recent years, a new subset of CD4^+^ T-cells has been discovered that helped to explain many of the discrepancies in the classic Th1/Th2 model, and it has been termed “Th17” based on its secretion of the novel proinflammatory cytokine IL-17 [23]. Cardoso et al. have demonstrated the presence of Th17 cells in the sites of chronic inflammation in human periodontal disease. They collected gingival and alveolar bone samples from healthy patients and patients with chronic periodontitis and demonstrated elevated levels of IL-17, TGF-*β*, IL-1*β*, IL-6, and IL-23 messenger RNA and protein in diseased tissues as well as the presence of Th17 cells in the gingiva from patients with periodontitis. Moreover, IL-17 and the bone resorption factor RANKL were abundantly expressed in the alveolar bones of diseased patients, in contrast to low expression level in controls [24]. Ohyama et al. have also reported that IL-17 is involved in periodontitis and the IL-23/IL-17 pathway is frequently induced in periodontitis lesions and that this pathway may therefore play an essential role in periodontal biology [25].

The role of IL-17 in periodontal disease is controversial. Whereas elevated IL-17 levels have been found in humans with severe periodontal disease [26], Yu et al. have recently reported that female C57BL/6J mice lacking the IL-17 receptor (IL-17RAKO) are significantly more susceptible to periodontal disease bone loss as a result of defects in the chemokine-neutrophil axis [27]. Further study demonstrates a gender-dependent effect of IL-17 signaling and indicates that gender differences should be taken into account in the preclinical and clinical study [28].

It is worth noticing that Th17 cells do not induce osteoclastogenesis in the absence of osteoblasts, which strongly suggests that RANKL expressed on Th17 cells alone is not sufficient to induce osteoclastogenesis: this is partly because Th17 cells produce a small amount of IFN-*γ*, which counterbalances the RANKL action [29].

#### 2.3.3. Treg Cells and RANKL Expression in Periodontal Tissues

The percentage of Foxp3^+^ cells is as low as 5% within the otherwise massive infiltration of RANKL^+^ lymphocytes found in the diseased gingival tissues. In the peripheral blood lymphocytes can be stimulated with bacteria (*A. actinomycetemcomitans*) in an antigen-dependent fashion; however, mRANKL expression is expressed prominently in Foxp3 negative cells and in Foxp3^dim⁡^ cells, not in Foxp3^bright^ cells, which most probably represent the presence of CD25/Foxp3 double-positive cells. IL-10 suppressed both sRANKL and membrane RANKL (mRANKL) expression by peripheral blood mononuclear cells (PBMC) activated *in vitro* in a bacterial antigen-specific manner. Taken together, these results suggest that Foxp3/CD25 double-positive Treg cells may play a role in the downregulation of RANKL expression by activated lymphocytes in periodontal disease tissues. These results lead to the conclusion that the phenomenon of diminished CD25^+^Foxp3^+^ Treg cells appears to be associated with the increased RANKL^+^ T cells in the bone resorption lesions of periodontal disease [30].

### 2.4. B-Cells and RANKL Expression in Periodontal Tissues

More than 90% of B cells recovered from human periodontal diseased tissues express RANKL, as opposed to about 54% of T cells [10]. B cells do not seem to require the presence of T cells to drive bone resorption. In a congenitally athymic rat model of experimental periodontitis injected with donor B cells, RANKL expression and the corresponding induction of osteoclast differentiation increased in rats receiving B cells from *A. actinomycetemcomitans*-immunized animals compared to nonimmune B cells [31]. In a recent study, it is suggested that RANKL expression is upregulated in B cells in the adaptive immune response rather than in the innate immune response to *A. actinomycetemcomitans*, and preimmunization of animals with *A. actinomycetemcomitans* leads to an enhanced B-cell response including increased RANKL expression [32]. A recent *in vitro* study indicated that toll-like receptors (TLRs) may play a role in B cell-mediated RANKL-dependent periodontal bone resorption, and TLR4 and TLR9 diminish RANKL production, probably through the induction of RANKL-expressing immune B cell apoptosis [33].

### 2.5. Osteoblasts, Osteocytes, and RANKL Expression in Periodontal Tissues

Mice with RANKL deficiency in osteoblast lineage have showed some protection from bone loss induced by ovariectomy as well as from joint destruction associated with arthritis, whereas loss of RANKL in T cells did not confer such protection, which indicated that RANKL expression by osteoblast lineage plays an important role in bone resorption [34]. Atkins et al. reported that RANKL expression was related to the differentiation state of human osteoblasts [35] and RANKL was expressed preferentially by immature osteoblasts and the expression level decreased during their maturation.

The idea that osteoblasts, or their progenitors, support osteoclast formation by expressing the cytokine RANKL is a widely held tenet of skeletal biology. But more recently studies provide evidence that osteocytes, and not osteoblasts or their progenitors, are the major source of RANKL driving osteoclast formation in trabecular bone. Nakashima et al. have reported that purified osteocytes express a much higher amount of RANKL and have a greater capacity to support osteoclastogenesis *in vitro* than both osteoblasts and bone marrow stromal cells. Furthermore, the severe osteopetrotic phenotype that they observed in mice lacking RANKL—specifically, in osteocytes—indicates that osteocytes are the major source of RANKL in bone remodeling *in vivo* [36]. However, femurs in mice lacking RANKL in osteocytes have normal shapes, indicating that modeling of the metaphyseal cortex of long bones is controlled by cells other than osteocytes. Thus, the role of osteocyte-derived RANKL may be limited to bone remodeling [37].

Given the special anatomy of periodontal tissue, the role of osteoblasts and osteocytes in periodontal diseases may be different from other bone resorption diseases, because osteoclasts are formed at different skeletal sites for different purposes. The results of the conditional RANKL deletion studies show that the osteoclasts that form at these different sites require different support cells in each case [37]. Specifically, the finding that osteocyte-derived RANKL is not required for tooth eruption or resorption of calcified cartilage during endochondral bone formation shows that other cell types must supply the RANKL required for osteoclast formation in these processes [38].

### 2.6. Macrophage and RANKL Expression in Periodontal Tissues

Although macrophage may be not the main source of RANKL expression in periodontal disease [10], it can influence RANKL expression through its pattern recognition receptors (PRRs) and cytokines [39].

#### 2.6.1. Macrophage PRRs and RANKL Expression in Periodontal Tissues

Macrophages express a lot of PPRs, such as TLRs, to recognize periodontal pathogens, and then induce a series of intracellular signaling events, NF-*κ*B activation, and culminating in expression of inflammatory mediators [39, 40]. Interestingly, the number of TLR2-expressing cells, but not of TLR4-expressing cells, tends to increase linearly with gingival inflammation. This may have to do with the fact that most suspected periodontal pathogens preferentially activate TLR2 rather than TLR4. Indeed, *P. gingivalis*, *T. forsythia*, *T. denticola*, *Prevotella intermedia*, *Prevotella nigrescens*, *Capnocytophaga ochracea, A. actinomycetemcomitans*, *Fusobacterium nucleatum,* and *Veillonella parvula* can all activate TLR2, but only the last three can efficiently activate TLR4. The regulated expressions of TLRs in the periodontium and their activation by periodontal bacteria suggest that TLRs are potentially major players in periodontitis. Whether TLRs are involved in protective immunity or destructive inflammation (or both) has yet to be elucidated. Their potentially ambivalent role may be reflected in the studies that aim to correlate single nucleotide polymorphisms of TLR genes with susceptibility to periodontitis, which have been inconclusive when taken together [41]. Clinical and experimental studies have identified that PRR-dependent recognition of *P. gingivalis* is an initial step in host response to this organism, and TLRs have emerged as a major group of PRRs involved in recognition and signaling in the context of *P. gingivalis* exposure. Indeed, experimental studies have identified that signaling through TLR2 leads to oral bone loss in mice [42].

It has been reported that the innate immune response promoted osteoclastogenic activity by activating RANKL via TLR pathways [43]. Others reported that innate immune recognition through TLR signaling is crucial for inflammatory bone loss in response to infection by microorganisms associated with chronic periodontal disease [44]. In a previous study, Rosen et al. showed that *T. denticola* lipooligosaccharide (LOS) produced a concentration-dependent activation of NO and TNF-*α* in murine macrophages which was inhibited by polymyxin B [45]. In their later study, they showed that this activation is dependent on the TLR4-MyD88 signaling pathway [46].

Despite these reports, clinical studies employing a genetic polymorphism approach are not in agreement regarding major roles for TLRs in periodontal disease. Schröder et al. [47] reported that a TLR4 polymorphism is associated with periodontitis, while Folwaczny et al. [48] failed to observe associations for TLR2 or TLR4. Thus it is likely that in addition to TLRs, other unrecognized PRRs may contribute to periodontal disease and host response to periodontal pathogens. Baer et al. reported that Scavenger receptor A is expressed by macrophages in response to *P. gingivalis* and participates in TNF-*α* expression [49], which can upregulate RANKL expression [50].

#### 2.6.2. IL-1, TNF-*α*, and RANKL Expression in Periodontal Tissues

TNF-*α* potently increased osteoclast proliferation/differentiation in the presence of RANKL. This effect was greatest when RANKL was present before exposure of osteoclast precursor cells to TNF-*α*. The resorptive activity of osteoclasts generated by TNF-*α* in the absence of RANKL was critically dependent upon IL-1, which was expressed by lymphocyte-monocyte interaction [51]. Further study indicated that IL-1 and LPS stimulate osteoclastogenesis through two parallel events: direct enhancement of RANKL expression and suppression of OPG expression, which is mediated by PGE2 production [52].

In the process of periodontal disease, a key role for TNF-*α* and IL-1 has been demonstrated, including regulation of osteoclastogenesis *in vitro* and *in vivo* [10, 50, 53–55]. It is noteworthy that during the progression of experimental periodontitis, high levels of IL-1*β* and TNF-*α* have been positively related to RANKL expression [56, 57]. Kawai et al. have also reported that the concentrations of sRANKL and IL-1*β* examined in the gingival tissue homogenates were significantly elevated in the diseased gingival tissues compared to healthy tissues [10]. Wei et al. reported that IL-1 mediates the osteoclastogenic effect by enhancing stromal cell expression of RANKL and directly stimulating differentiation of osteoclast precursors [50]. Fujihara et al. reported that TNF-*α* enhances RANKL expression in gingival epithelial cells via protein kinase A signaling [58].

### 2.7. Periodontal Ligament Fibroblasts, Gingival Fibroblasts, and RANKL Expression

It had been reported that human periodontal ligament cells stimulated with LPS inhibit osteoclastogenesis by producing more effective OPG than RANKL through the induction of IL-1*β* and TNF-*α* [59]. Further studies indicated that IL-1*α* stimulates osteoclast formation by increasing the expression level of RANKL versus OPG via ERK-dependent PGE2 production in PDL cells [9]. But a more recent study reported that OPG was detected at high levels in both fibroblast cultures, whereas RANKL could not be detected [60].

Resorption of bone did not occur by the mononuclear cells (MNCs) formed in the presence of fibroblasts, suggesting that fibroblasts may secrete inhibitors for bone resorption, which leads to the osteoclast-like cells dysfunction. The incapacity of the MNCs to resorb bone under the influence of fibroblasts can be reversed by adding macrophage colony-stimulating factor (M-CSF) and RANKL in the culture media. These results suggest that tooth-associated fibroblasts may still be able to trigger the formation of osteoclast-like cells, but more importantly, they play a role in preventing bone resorption, since additional stimuli are required for the formation of active osteoclasts [60].

## 3. Conculsions 

**Figure 1:**
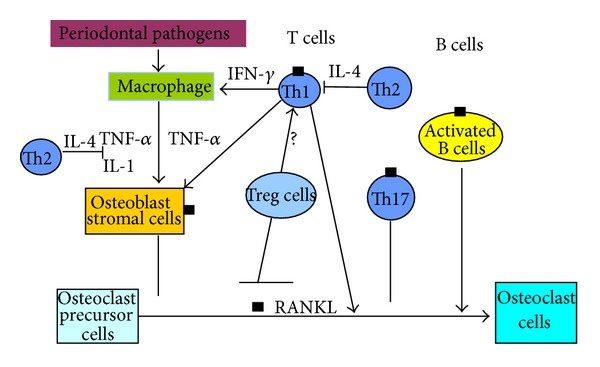
B cells and T cells are primary RANKL expression cells in periodontal bone resorption. Th17 cells can express RANKL; however, RANKL expressed on Th17 cells alone is not sufficient to induce osteoclastogenesis. Treg cells may play a role in the downregulation of RANKL expression by activated lymphocytes in periodontal disease tissues, but the mechanism is unclear. Osteoblast stromal cells may also express RANKL, but they play a role in preventing bone resorption, since additional stimuli are required for the formation of active osteoclasts. Although macrophage may not be the main source of RANKL expression in periodontal disease, it can influence RANKL expression through its cytokines.

RANKL expression in periodontal tissues is a very complicated process that involves many factors. RANKL is identified in lymphocytes, stromal cells, and many other cell types in periodontal tissues which play an important role in direct or indirect regulatory roles. Cytokines such as IL-1*β* and TNF-*α* can upregulate RANKL expression in periodontal cells and increase osteoclast formation.  Figure 1 summarizes the major cells and cytokines related to RANKL expression.

Therefore, discovering the pivotal step in RANKL expression may lead to a new insight into periodontal pathogenesis and the development of a new target for periodontal therapy; for example, we can prevent periodontal bone resorption through RANKL expression inhibiting by moderating lymphocytes' function or changing some cytokines' level.
